# COVID-19 Contact Tracing Using Blockchain

**DOI:** 10.1109/ACCESS.2021.3074753

**Published:** 2021-04-21

**Authors:** Haya R. Hasan, Khaled Salah, Raja Jayaraman, Ibrar Yaqoob, Mohammed Omar, Samer Ellahham

**Affiliations:** 1 Department of Electrical Engineering and Computer ScienceKhalifa University of Science and Technology 105955 Abu Dhabi United Arab Emirates; 2 Department of Industrial and Systems EngineeringKhalifa University of Science and Technology 105955 Abu Dhabi United Arab Emirates; 3 Heart and Vascular Institute, Cleveland Clinic Abu Dhabi Abu Dhabi United Arab Emirates

**Keywords:** COVID-19, blockchain, Ethereum smart contracts, transparency, security analysis, contact tracing

## Abstract

Contact tracing has widely been adopted to control the spread of Coronavirus-2019 (COVID-19). It enables to identify, assess, and manage people who have been exposed to COVID-19, thereby preventing from its further transmission. Today’s most of the contact tracing approaches, tools, and solutions fall short in providing decentralized, transparent, traceable, immutable, auditable, secure, and trustworthy features. In this paper, we propose a decentralized blockchain-based COVID-19 contact tracing solution. Contact tracing can greatly suffice the need for a speedy response to a pandemic. We leverage the immutable and tamper-proof features of blockchain to enforce trust, accountability, and transparency. Trusted and registered oracles are used to bridge the gap between on-chain and off-chain data. With no third parties involved or centralized servers, the users’ medical information is not prone to invasion, hacking, or abuse. Each user is registered using their digital medical passports. To respect the privacy of the users, their locations are updated with a time delay of 20 minutes. Using Ethereum smart contracts, transactions are executed on-chain with emitted events and immutable logs. We present details of the implemented algorithms and their testing analysis. We evaluate the proposed approach using security, cost, and privacy parameters to show its effectiveness. The smart contracts code is publicly made available on GitHub.

## I. Introduction

The year 2020 has witnessed the spread of Coronavirus-2019 (COVID-19) all over the world. The major surge of the virus globally shook the foundations of the health sector which weren’t adequately prepared and couldn’t respond with high efficiency. Hence, the advancements in technology can help in restoring human lives across the world. In efforts to mitigate the unprecedented spread of COVID-19, contact tracing applications have widely been developed [1]–[3]. Contact tracing applications are believed to be able to break the chain of COVID-19 infections [4]. Contact tracing or proximity tracing can be used to identify how close someone has been to other contacts regardless of the context and one of those people in contact is positive to the pandemic virus. Tracing back all the possible contacts to the positive case and informing them about the possibility of being infected is part of contact tracing. This is essentially important to stop the further spreading of COVID-19.

The deficits of the current contact tracing technologies and solutions should be overcome to make contact tracing more successful. Three main issues; namely, privacy, accountability, and transparency must be considered when developing contact tracing applications [5]. Privacy of the users should be maintained where the data of users and their personal information should not be stored on centralized servers that are prone to hacking and abuse. On the other hand, accountability refers to the role of the technology held accountable for the decisions taken based on the lack of precision in proximity measurements. Additionally, transparency is vital when trying to communicate with the individuals of the societies. Application users should know how their input is being used, how it is processed, and where is the output used and distributed. However, the current systems do not consider the privacy of the users and are not transparent enough. Hence, users are discouraged from using the contact tracing applications [6].

In this paper, we aim to curb the spread of COVID-19 infections through a blockchain-based contact tracing solution. We leverage the use of the intrinsic features of blockchain technology to deal with the contact tracing challenges. Therefore, the implemented solution respects the privacy of its users. It is immutable, transparent, and accountability is a built-in feature by design. Blockchain is a distributed shared ledger that is decentralized with tamper-proof and immutable logs [7]. It is a linked list where all nodes keep a local copy of all the nodes [8]. Blockchain has a wide range of applications from e-commerce, to supply chain management [9]. In the context of the COVID-19 pandemic, blockchain has proven to be a versatile technology that can be used in several applications to mitigate the spread of infections [10], [11].

Contact tracing is one of the many ways that blockchain has proved to be useful to eradicate the effects of the pandemic. Using Ethereum blockchain with the added programmable logic using smart contracts allows the different participants to be transparent, accountable, and trusted. Our solution eliminates third-party servers, centralization, and identity abuse. It relies on the distributed ledger’s immutable logs to enforce transparency and trust. All transactions taking place on-chain are signed by their creator. Hence, every on-chain participant is held accountable for their action.

### A. Related Work and Contributions

Herein, we review the existing literature available on contact tracing applications and their integration with blockchain.

The authors in [12] presented a survey on contact tracing applications used for COVID-19. The authors were aimed to highlight the key differences and features of the different applications especially in certain attributes related to the system architecture and design, data security and privacy as well as attacks and vulnerabilities. The authors outlined different applications such as Trace Together, Covid Safe, Covid Watch, and EpiOne. The authors concluded that each application based on its architecture whether centralized, decentralized, or hybrid has its pros and cons. They also emphasized on the adoption rate between the users and how it can increase with transparency. Decentralization in their context referred to reducing the load on the servers and increasing it on the user devices. They did not tackle the aspect of decentralization using blockchain-based solutions.

The authors in [11], [13] discussed that blockchain can be used to create a decentralized contact tracing solution. Its intrinsic features help in ensuring transparency, trust, pseudo-anonymity, and decentralization. In [14], the authors presented a blockchain-based contact tracing framework. They highlighted the privacy concerns in the available contact tracing applications due to the use of centralized servers and suggested a blockchain-based design that could overcome the privacy issues of the existing solutions. Their solution does not show any implementation details or testing results. On the other hand, BeepTrace [15] emphasized on the importance of using blockchain in contact tracing to add trust, transparency, and privacy. Hence, in their solution, they introduce two types of blockchain networks one for the tracing and the other one for the notifications. BeepTrace shows promising results when compared to other solutions presented by the authors in terms of cost, security, and privacy. However, its solution depends on using trusted third parties such as the ‘Geodata solvers’ servers as well as a Public Key Infrastructure (PKI).

To overcome the use of third parties, the authors in [16] proposed a blockchain network named Bychain. In the network, they amend the fields of the used blockchain blocks to suit their block proposal and validation scheme. Their solution also depends on short-range communication (SRC). The testing is done on the implemented blockchain network design to test the messages’ latency, power consumption, computation, and storage limitations. Their main objective is to test the newly created Bychain which offers limited information on results related to COVID-19 contact tracing. Moreover, the study conducted in [17] made use of an Internet of Things (IoT) hardware model that uses passive RFID transceivers. The solution ensures that users remain anonymous until a user tests positive for the COVID-19 virus. The study presents smart contract (SC) codes, as well as hardware details. However, the study does not present a complete architecture or design of the proposed blockchain solution. Furthermore, the study conducted in [18] presents a contact information sharing and risk notification system using blockchain. Their solution uses Bluetooth as a short-range communication technology. Their solution concentrates on quantifying the possibility of a user infection based on the information provided by the user or from the shared information. The status of the user changes based on information entered by the users. The possibility of infection for other contacts is calculated using an equation proposed by the authors.

**FIGURE 1.**
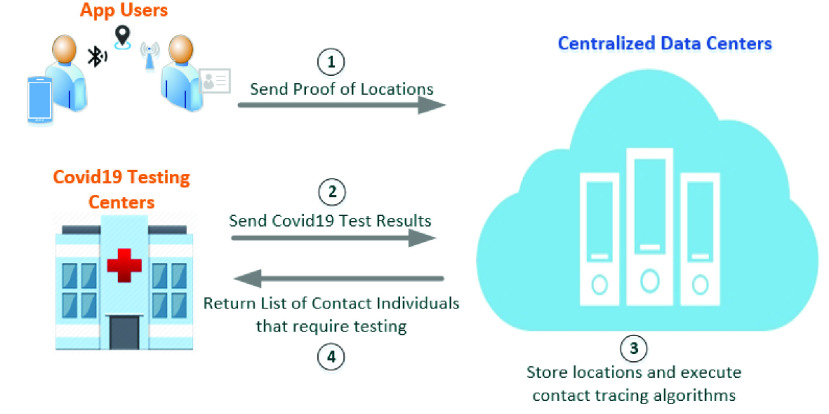
Centralized contact tracing applications using servers.

Numerous research efforts have been conducted on COVID-19 contact tracing [12]. However, most of the existing solutions are based on centralized servers or third parties [19]. Figure 1 presents a basic architecture of the centralized solution that depends on servers. The application users send their location information to centralized data centers. On the other hand, the COVID-19 testing centers send the COVID-19 test results to the centralized servers. Such servers do all the processing based on the input received by the application users and the testing centers. The result containing the COVID-19 contacts list is then sent to the COVID-19 testing centers to take the needed action. Hence, in such an architecture, the users’ information is all stored on the servers. Also, there is no transparency, and everyone involved needs to trust the server and each other in terms of honesty. Furthermore, such a system is prone to the single point of failure problem. Unlike the aforementioned solutions, our solution leverages blockchain technology and utilizes its built-in security features to present transparent, tamper-proof, and immutable transactions between all participating entities.

The main contributions of this paper can be summarized as follows:

• We showcase a blockchain-based approach to enable and provide COVID-19 contact tracing in a manner that is fully decentralized, transparent, traceable, immutable, auditable, secure, and trustworthy.
• We integrate the Ethereum blockchain with on-chain registered oracles to execute the contact tracing algorithms to put down the extra burden and save cost.
• We develop smart contracts along with algorithms to implement functionalities and define rules regarding COVID-19 contact tracing applications. The smart contracts code is publicly made available on GitHub.^1^
• We perform the cost analysis to show feasibility and affordability of the solution, and present security analysis to show that our smart contracts are secure enough against well-known vulnerabilities and attacks.
• Our proposed blockchain-based COVID-19 contact tracing solution is generic and can be easily customized as per the needs and requirements of various types of contact tracing applications focusing on other diseases.

^1^https://github.com/smartcontract694/ContactTracing/blob/main/Code

The rest of the paper is organized as follows. Section II presents the design details of the proposed blockchain-based solution followed by the implementation details in section III including the smart contracts and algorithms. Section IV presents the testing details of the proposed system followed by section V which showcases the security and cost analysis along with discussing the privacy and generalization aspects. Section VI concludes the paper.

## II. System Design

**FIGURE 2.**
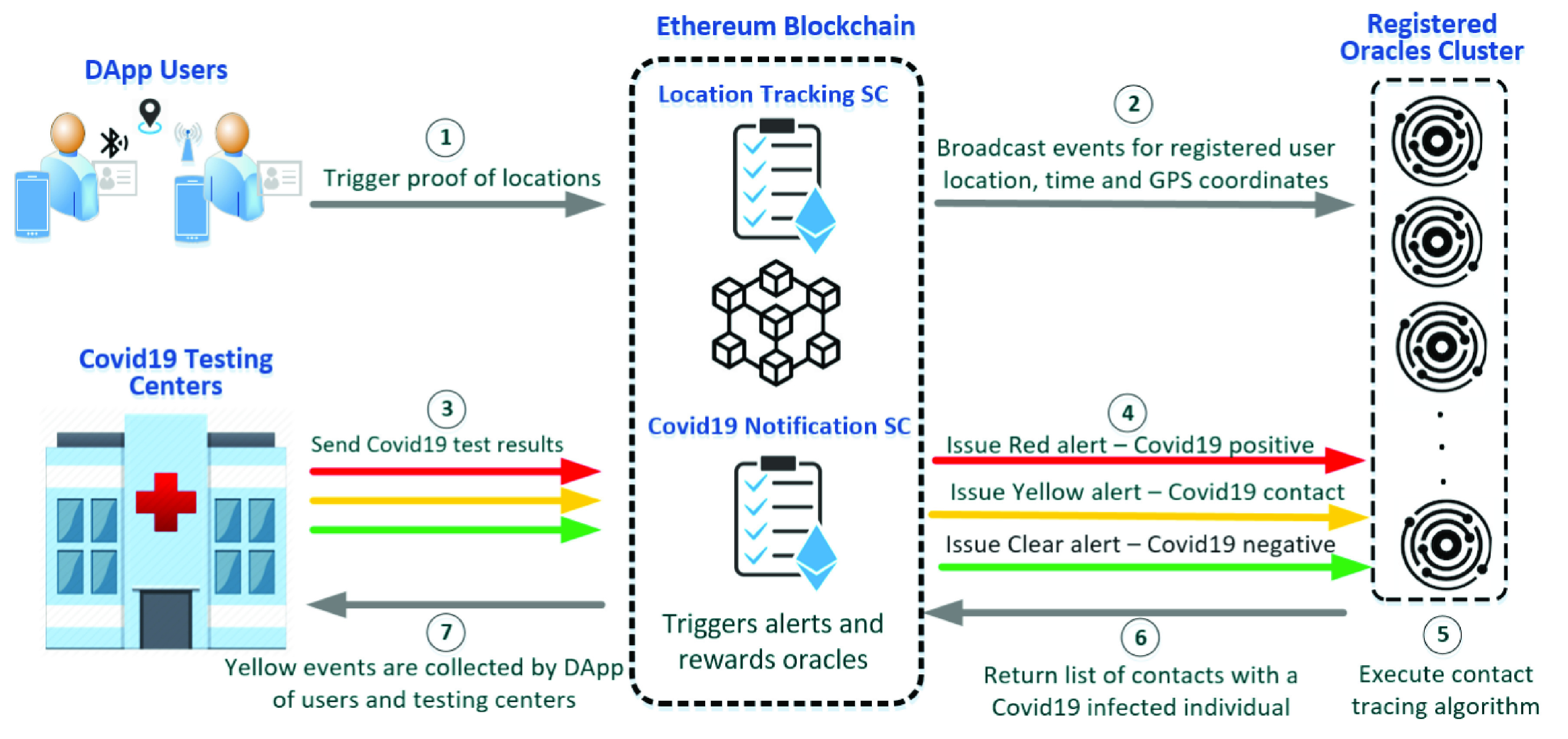
System diagram of the proposed blockchain-based COVID-19 contact tracing solution.

This section presents the system design of our proposed blockchain-based COVID-19 contact tracing solution. Figure 2 shows the system components of the solution along with the interacting entities. The decentralized application (DApp) users employ their smartphones to trigger proof of locations that are logged on-chain through the smart contracts. Consequently, the registered oracles use the broadcast events and locations in their contact tracing algorithms. On the other hand, the COVID-19 testing centers send COVID-19 results to the blockchain to trigger alerts as needed. The alerts issued vary depending on the received result. Those emitted events are alerts used to notify the registered oracles to execute the contact tracing algorithm. The registered oracles return the list of contacts of the possibly infected individuals to the smart contract. There are no centralized servers involved, thereby overcoming the burden used to pose traditional centralized systems. This also adds a layer of trust, transparency, and integrity.

### A. DApp Users

DApp users employ the downloaded contact tracing application to trigger proof of locations. Those proof of locations have the latitude, longitude, time, and date that are communicated to the ‘Location Tracking SC’. Communication happens when a user is within contact with another user at a distance of fewer than 2 meters only. This allows saving on power and cost. Smartphones find each other through short-range communication such as Bluetooth. Other means are also possible using WiFi or mobile data. The distance is calculated using the geo-location data exchanged between two users in close proximity. If the distance is less than 2m, the proof of locations should be logged on-chain. The distance between two geopoints can be calculated using the Haversine formula [20], [21]. This formula finds the distance between two points on a sphere using their latitudes and longitudes. Each user’s proof of location and contact details are stored on the ledger in a tamper-proof manner. Furthermore, there is a 20 minute delay before sending proof of location to preserve the privacy of the user. This ensures that the current location of the user is not known. Hence, the user’s privacy is not abused through invasion.

The application users must be registered to use the contact tracing application and on-chain functions. The registration is done through the affiliated testing centers and every Ethereum address (EA) is associated with bio-metrics data. Furthermore, the registered users have a digital medical passport that has all their medical information [22]. Health Passport is identified as an emerging technology in Gartner’s 2020 Hype Cycle [23] and this research uses the concept of the medical passport from our previous work conducted in [22] as part of the solution. The users’ right to convey the information included in their medical passports to other entities is based upon their choice [22]. All the information on-chain is stored using IPFS hashes. The information is kept confidential until the user allows a certain entity to get access to the information. Hence, only then this information is disclosed to the entity authorized by the user [22]. Therefore, authorization is needed by the users to disclose any of their information.

### B. COVID-19 Testing Centers

COVID-19 testing centers are specialized in conducting COVID-19 tests. They would communicate the result with the ‘COVID-19 Notification SC’ to issue an alert based on the result. The COVID-19 testing centers also await a response from the COVID-19 Notification SC. This response helps them in finding out the people of contact that need to be quarantined, tested, and treated if needed. The SC in response issues yellow alerts that are based on the result of the contact tracing algorithms run by the registered oracles. Hence, the COVID-19 testing centers are the point of contact between the test takers and the COVID-19 Notification SC.

### C. On-Chain Smart Contracts

Using the Ethereum blockchain, it is possible to execute smart contracts that carry programmable logic. Digital assets as well as ‘Ether’ can be controlled using the logic presented in the SC. Events are emitted in logs to ensure that the actions taken are transparent and immutable. This establishes trust among all the participating entities on-chain. In our proposed solution, we have implemented two smart contracts.

#### 1) Location Tracking SC

In the location tracking SC, locations of the DApp users are logged. This is the main task of the smart contract. This is a very vital step to ensure that all contacted individuals can be traced based on their locations. The decentralized applications communicate to the blockchain regarding the locations of their users through the location tracking smart contract. The longitude, latitude, as well as time, are all sent to be immutably logged. The smart contract, on the other hand, can emit events that get broadcast to all the registered oracles as well as participating entities. This ensures that the GPS coordinates are available when needed for contact tracing.

#### 2) COVID-19 Notification SC

The COVID-19 notification smart contract handles COVID-19 alerts. The alerts are of three types based on the COVID-19 test results. Red, yellow, and green are the three colors used to identify the alert types. The different alerts are issued by the smart contract after the COVID-19 test results are sent by the COVID-19 testing centers. The issued alerts are then used to update the on-chain profiles of the users as well as alert the registered oracles of the new updates. The registered oracles use the logged COVID-19 alerts on-chain to know whether a contact list is required. A red or yellow alert based on the testing center results indicates that the contact tracing algorithm must be executed. However, it does not require to be executed in the case of a green alert. The registered oracles execute the contact tracing algorithm and return the list of contacts that could be infected. The individuals’ EAs are entered into the smart contract and the results are emitted as yellow alerts. Those alerts are used by the testing centers as well as DApp users to notify the users that they could be infected by the virus. The status of the users would turn to yellow based on the contact tracing information.

### D. Registered Oracles

Oracles are used to connect the blockchain to the off-chain data and input. To maintain the deterministic nature of the decentralized blockchain, no external calls to any APIs are allowed. Therefore, oracles are the bridges used to connect the blockchain to the outside world; hence, named as blockchain middleware [24].

#### 1) The Oracle Problem

The oracle problem is one of the common problems in the Oracle-based systems [25]. Choosing only one oracle as the main source of information from the outside world leads to create dependency on a single entity that can result in a single point of failure [26]. Furthermore, it is hard to assume that a single oracle can be trustworthy given the possibility that it can be hacked and depreciated. To solve the oracle problem, a network of oracles need to be used [27]. Hence, we used a cluster of registered oracles that communicate with the Ethereum smart contracts on-chain. This ensures that the blockchain has access to reliable secure off-chain data. Decentralized networks of oracles are a solution that maintains the distributed nature of blockchain at the same time along with ensuring that the blockchain can have access to real-world data and information.

#### 2) Oracles Registration

Oracles need to register so that they become authorized to access the functions in the smart contracts. In our design, the ‘Oracles SC’ is the smart contract responsible for the services of the oracle such as registration, awarding, and penalizing. The ‘Oracles SC’ owner is an authorized entity that would accept the registration of an oracle. Once an oracle’s Ethereum address is part of the registered oracles, it becomes authorized to execute the functions in the COVID-19 Notification smart contract. A previously registered oracle can also become prohibited from accessing or executing the functions. This is also done through the ‘Oracles SC’.

#### 3) Oracles Contact Tracing Results

**FIGURE 3.**
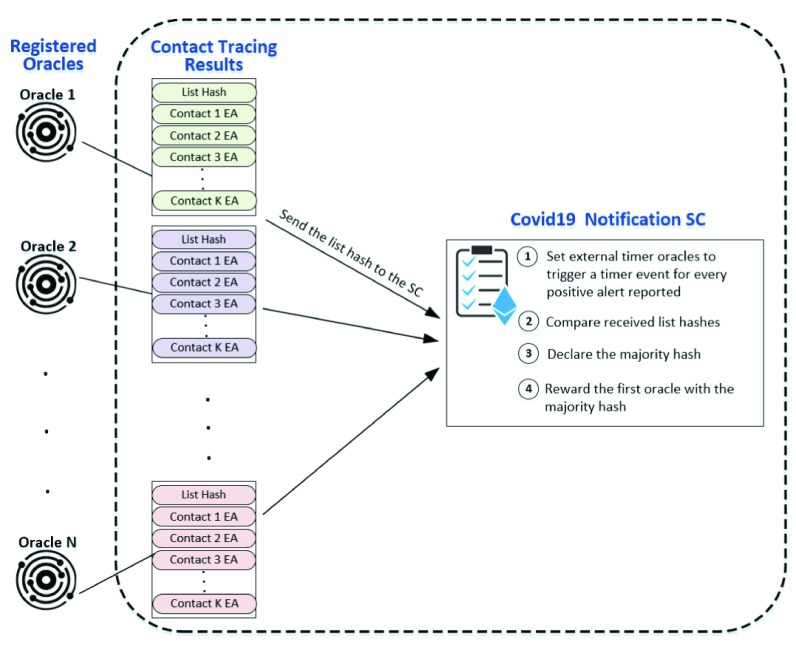
Oracles communication with the COVID-19 Notification SC.

The registered oracles execute the contact tracing algorithm in response to the received red or yellow alert from the ‘COVID-19 Notification SC’. Every alert sent by the ‘COVID-19 Notification SC’ possesses an ID to clearly label and identify it. The list produced by the oracles is assembled off-chain and its hash is computed. The list hash is then sent to the COVID-19 Notification SC. Each registered oracle needs to respond within the time allocated by the smart contract. The oracle has to send the list hash to the smart contract along with the request ID. Once the deadline is reached the timer oracle would then call the COVID-19 Notification SC to start comparing the received hashes and to declare the majority hash. The majority hash is the hash that matches at least 50 % of the compared hashes. Figure 3 shows the communication details between the registered oracles and the COVID-19 Notification CS. It illustrates how the oracles execute the contact tracing algorithm and then send the hash to the smart contract for comparison and further computations.

#### 4) Oracles Rewards and Penalty

Generally, malicious oracles can lead to leak users’ location information along with timestamp information. In addition, they can disseminate incorrect COVID-19 test results to create panic situations. Therefore, it is important to identify and penalize malicious or suspected oracles. Specifically, in our use case scenario, only an oracle whose result matches with the majority of oracles’ results is considered the winning oracle and is rewarded for its timely and accurate response. Oracles should be rewarded and penalized based on their behavior and output results [28]. This is done through digital assets, e.g., Ether and reputation systems. The reputation of oracles depends on how well they execute their tasks, their honesty, and their trustworthiness. Hence, each registered oracle is associated with a reputation that gets affected based on its performance. The winning oracle is rewarded with Ether to encourage other oracles to try and respond promptly. On the other hand, malicious oracles whose results do not match with the majority of oracles’ results are usually blacklisted through low rating and penalty mechanisms.

## III. Implementation Details

This section presents the proposed algorithms along with their implementation and coding details. The solidity code is written and tested using the Remix IDE [29]. Further discussion is provided in the following subsections.

### A. Proof of Locations

Algorithm 1 shows the details of the $TriggerLocationAlert$ function available in the ‘Location Tracking SC’. In this function, proof of locations of the mobile applications users is emitted by the smart contract based on the input received. The notification that is alerted contains the longitude, latitude, EA of the application user as well as the time.

**Algorithm 1:**
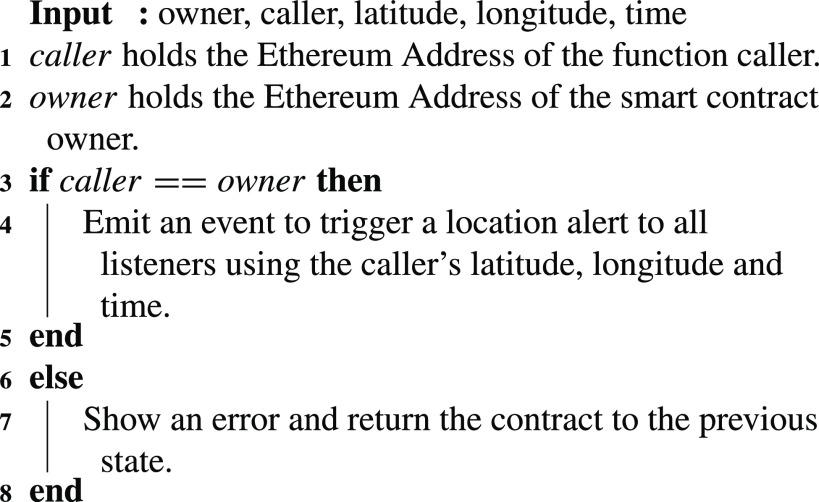
Proof of Locations

### B. Oracle Registration

The oracles that participate in the smart contracts must be registered. Algorithm 2 presents the registration details of the oracles in the ‘Oracles SC’. Oracle registration is only possible through the smart contract owner. Hence, the algorithm checks the EA of the function caller. Then a mapping is used to complete the registration where the EA of the oracle is mapped with the boolean ‘true’.

**Algorithm 2:**
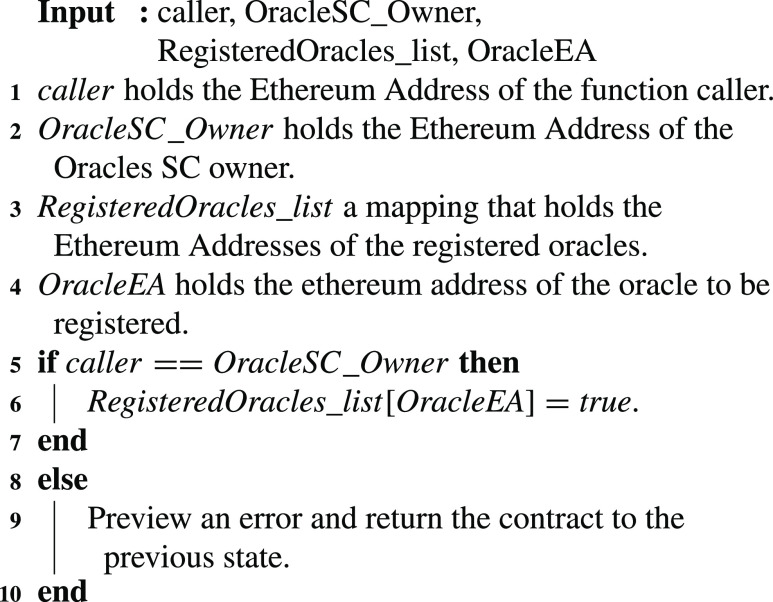
Oracle Registration

### C. Oracle Revoking

If an oracle can no longer participate with the smart contracts, then it is revoked by the Oracle SC owner. Algorithm 3 shows the details of revoking the previously registered oracle. This is done by mapping the EA of the oracle to a boolean value of ‘false’.

**Algorithm 3:**
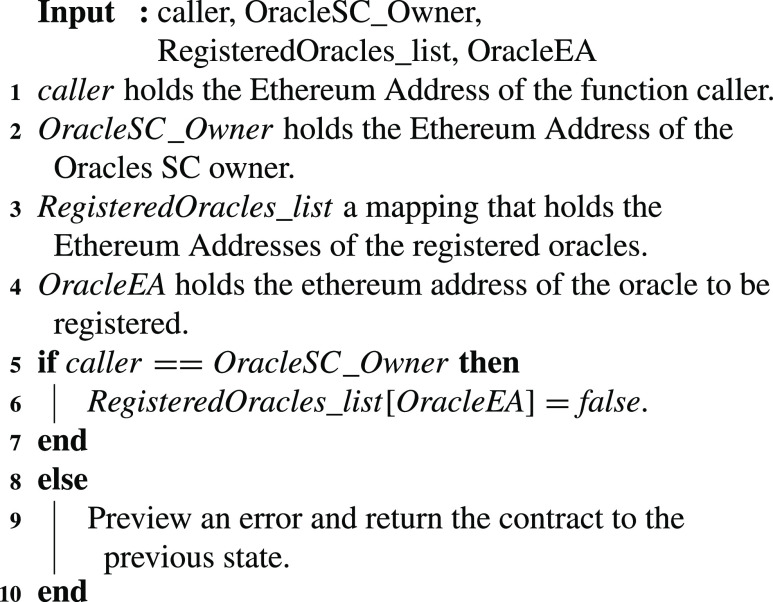
Oracle Revoking

### D. Broadcast COVID-19 Test Results

In the COVID-19 Notifications SC, the COVID-19 test results are sent by the testing centers to the smart contract. This is done through the $SendCovid19TestResults$ function. Algorithm 4 explains the details followed in the function. The results show three types of events either a test taker is COVID-19 positive, or a contact, or COVID-19 negative. Those results emit a red, yellow, or clear alert, respectively. In algorithm 4, the result is an unsigned integer. Hence, the values chosen to represent a positive, contact, or negative case are 1, 2, and 3 respectively. This saves on the cost of comparing strings. Comparing integers is much more cost-efficient in Solidity. Each result received by the testing center and being sent on-chain is given an ID. A red request ID and a yellow request ID are also maintained for tracing back easily when needed. The request ID is stored whenever a new request is created as can be seen in algorithm 4. This algorithm can only be executed by the smart contract owner who is the testing center representative on-chain. Therefore, a modifier is used to restrict access to the function accordingly.

**Algorithm 4:**
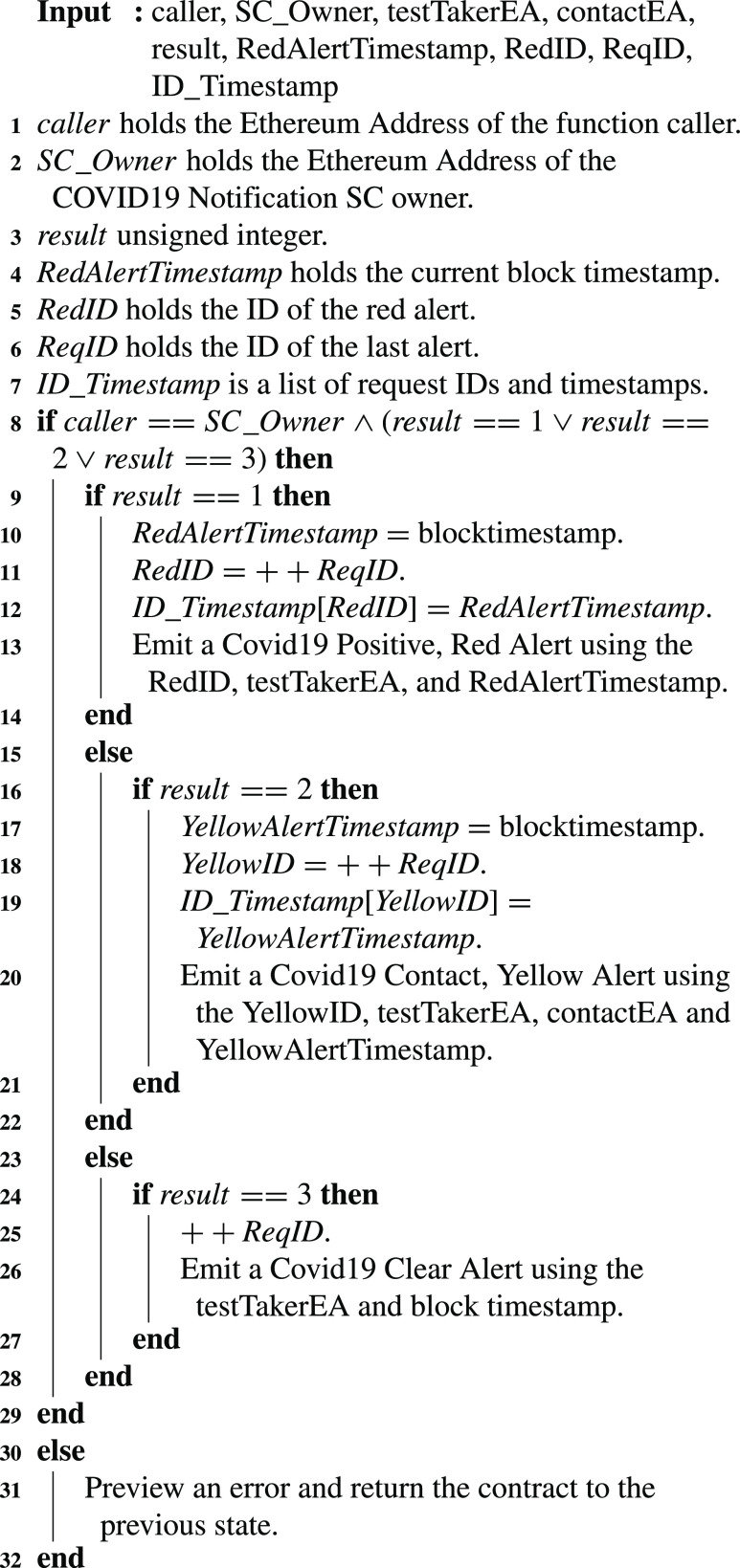
Broadcast COVID-19 Test Results

### E. Return the Contact Tracing List Hash

Once an event is emitted for a red or a yellow alert, the registered oracles’ role commences. Each of the registered oracles should execute the contact tracing list algorithm to find out the EAs of all the contacted people who could be infected by the COVID-19 virus. Those EAs could be numerous. Consequently, a hash is sent by the registered oracles using algorithm 5. Each registered oracle calls the function using the request ID, request timestamp, and the list hash. The function as can be seen in the algorithm checks the EA of the oracle to ensure the oracle is registered. Furthermore, it verifies that the ID and timestamp match the ID and timestamp of the request ID. In addition to those two aforementioned restrictions, the oracle has to reply within the allocated time of 60 seconds, otherwise, the entry is refused. Moreover, this function will only accept entries for a particular request ID at a time. Hence, it processes the request IDs sequentially. It first accepts all the entries for the current request ID and then once that request’s deadline approaches and the timer oracle ends the time for it, this function starts accepting entries for the next request ID. This is important to avoid making lists for each request ID at the same time which would cost a lot more compared to the current approach where only one list is reused every time a new request ID is processed. Five restrictions are important to ensure the proper execution of this function. Last but not least, the algorithm ensures that each oracle can only execute the function once. This is vital to ensure that it is fair for all the registered oracles. Also, this helps in mitigating the risk of having one oracle dominating and abusing their powers to deny others from casting their replies. Hence, to achieve this a unique value is stored every time an oracle submits its list hash. This unique value is the $keccak256$ hash of the oracle’s EA concatenated with the request ID.

**Algorithm 5:**
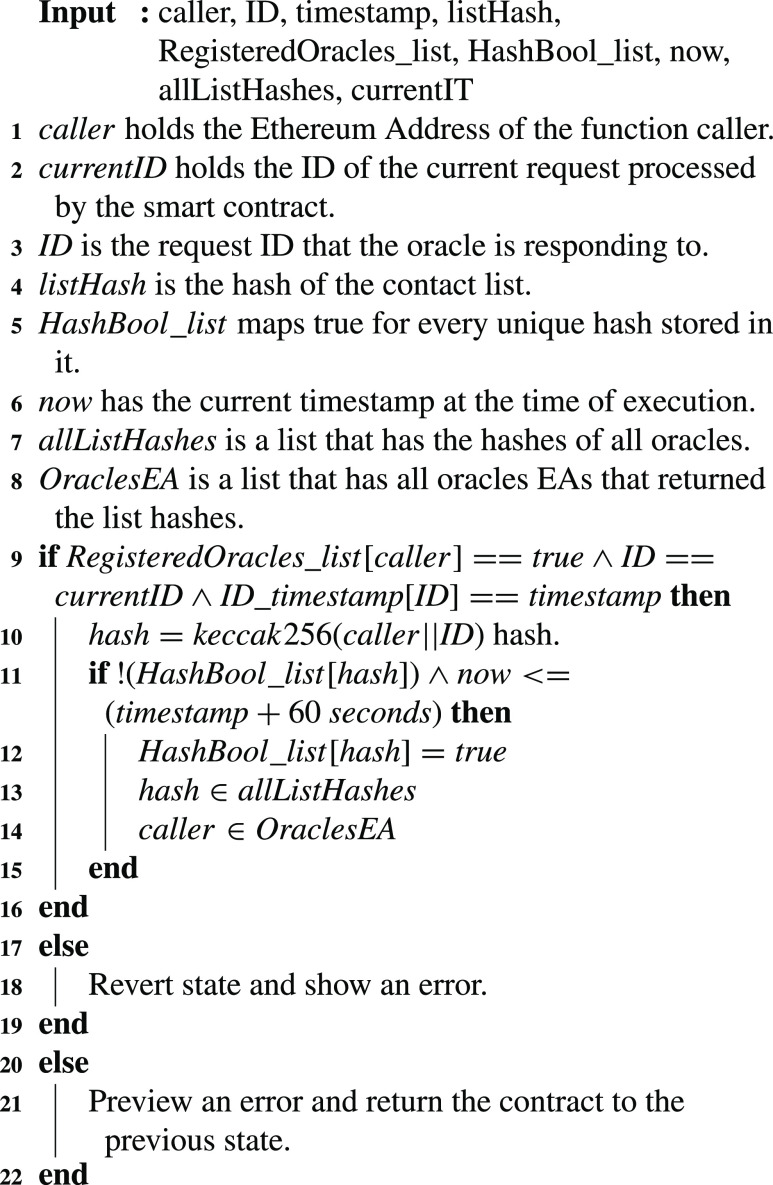
Return Contact Tracing List Hashes

We have used a combined index to ensure the value is unique for every entry submitted by an oracle. This combined index is then mapped to a boolean value. When an oracle returns a list for the first time, the combined index created is mapped to $true$. This value is checked every time an oracle returns a list. If the value returned is true, the check returns a $false$ and the contract state is reversed. The $keccak256$ hash is used instead of other hashes as it is the least expensive in terms of gas cost. To be able to easily find the hash of the oracle’s EA concatenated to the request ID using built-in solidity functions only, the $keccak256$ function of solidity accepts multiple values together if they are padded correctly. Hence, we ensured that no padding is done between both values when concatenated using the built-in function $abi.encodePacked$. This function concatenates both values in a byte array in the memory without changing their values. Consequently, in solidity we used the following line of code to hash and concatenate as needed $keccak256(abi.encodePacked(msg.sender,ID))$.

### F. Find Majority of the Contact List Hashes

Algorithm 6 describes the details of finding the most common hash (majority) of all the received list hashes. The smart contract finds out the most repeated hash of all the hashes submitted by the registered oracles. A maximum count is stored in the algorithm and is swapped when a new maximum is found. The majority value is concluded when the maximum count is equal to or exceeds half of the number of hashes submitted. The algorithm returns the index of the majority value as an output.

**Algorithm 6:**
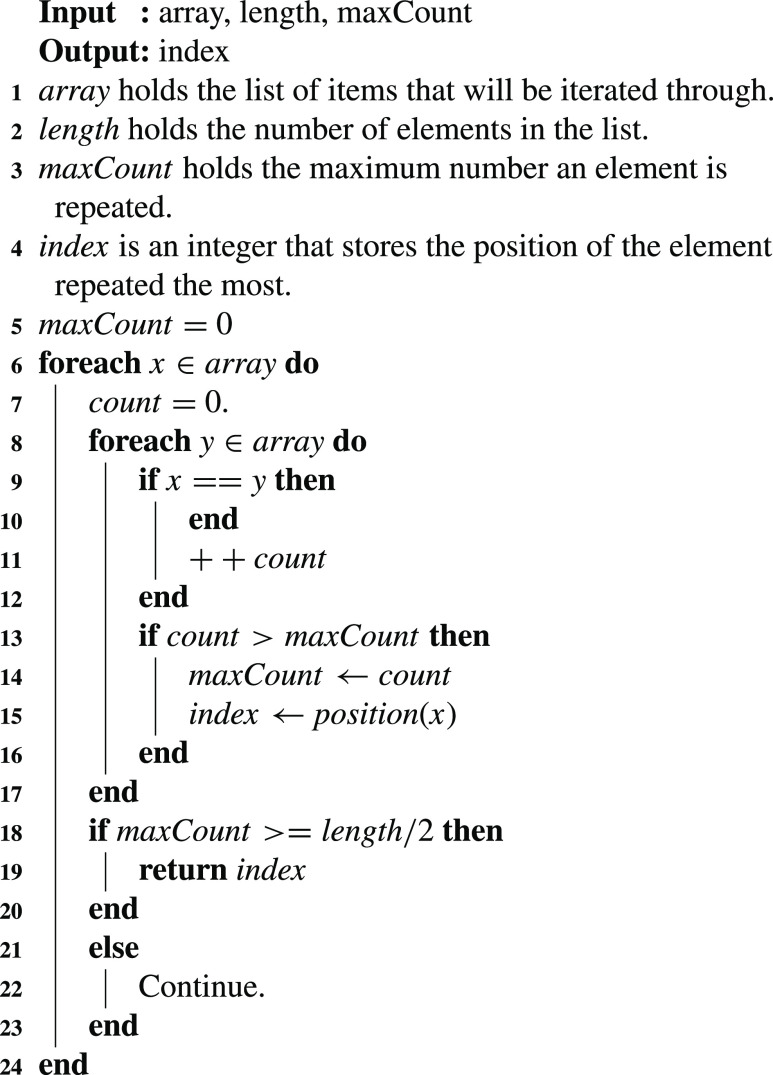
Find Majority

### G. Choose the Winning Oracle

In algorithm 7, the timer oracle executes a function when the allocated time for the oracles to submit their hashes is over. When the deadline for the oracles to submit their hashes is reached, algorithm 6 is called from within algorithm 7. The returned index from algorithm 6 is then used to locate the EA of the first oracle that replied with the chosen winning hash. A notification is emitted with the oracle’s EA and the winning hash. Once the winning oracle is chosen, the request ID that can now be handled by the smart contract is incremented by 1. Furthermore, all the arrays used for storing and choosing the majority hash and winning oracle for the previous ID are deleted to be ready for processing the new request.

**Algorithm 7:**
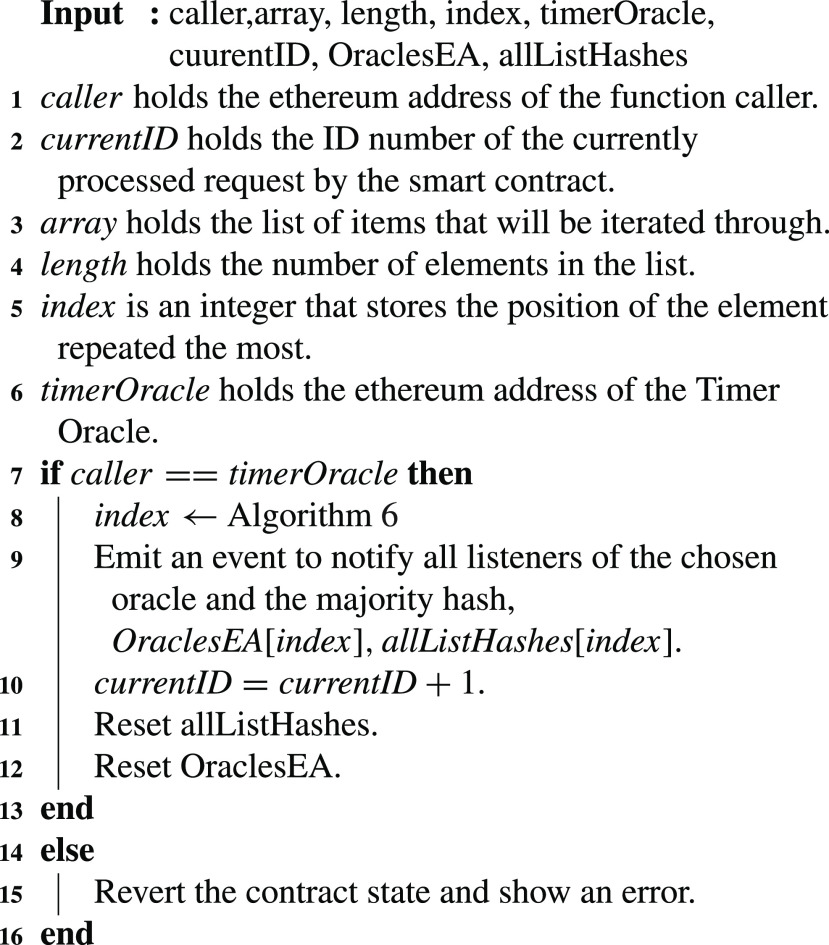
Choose Oracle

### H. Submit Contact Tracing List EAs

The selected winning oracle will then submit the EAs of all the individuals in the contact tracing list. This is done one by one by executing the function $EnterContactTracingList$ in the COVID-19 Notification SC. The function executes the algorithm described in algorithm 8 where the selected oracle can only be allowed to call the function. Furthermore, the EAs of the individuals are entered through the function as well as the request ID and a boolean value. The boolean is used to indicate if there are more function calls to be executed for the same request. For instance, when the chosen oracle reports the last EA, the boolean value reported would be false, unlike the previous calls where it was true. Whenever the function is executed, an event is emitted to issue a yellow alert introducing the individuals of the list using their associated EAs.

**Algorithm 8:**
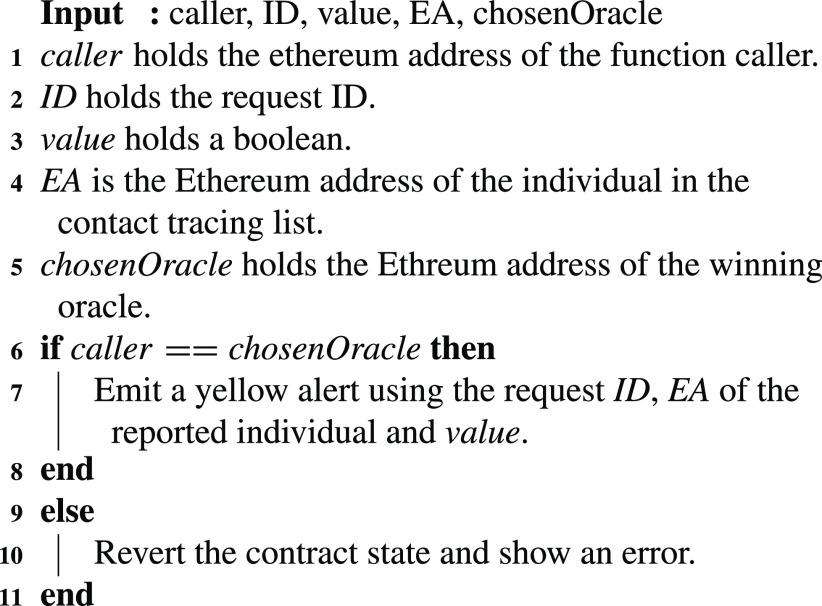
Submit Contact Tracing EAs

## IV. Testing and Validation

In this section, we rigorously tested the proposed smart contracts and presented their results. In our testing, the functions are tested for their functionality as well as the restrictions on them. Each function with a modifier to restrict the identity of the executor is tested with other EAs and the result is verified. Moreover, events and their logs are checked to ensure that they are as expected. Each smart contract has an owner. The owner could be a predefined EA in the smart contract or the EA of the entity that deploys the smart contract to the blockchain. The participating entities that interact with the smart contracts are the Location Tracking SC owner, the Oracles SC owner, the Notification SC owner, the registered algorithm tracing oracles, and the timer oracle. The functions are executed using the Remix [29] IDE and the results are shown in snapshots that showcase the function executed along with the results.

### A. Location Tracking SC: Location Alert Triggering

**FIGURE 4.**
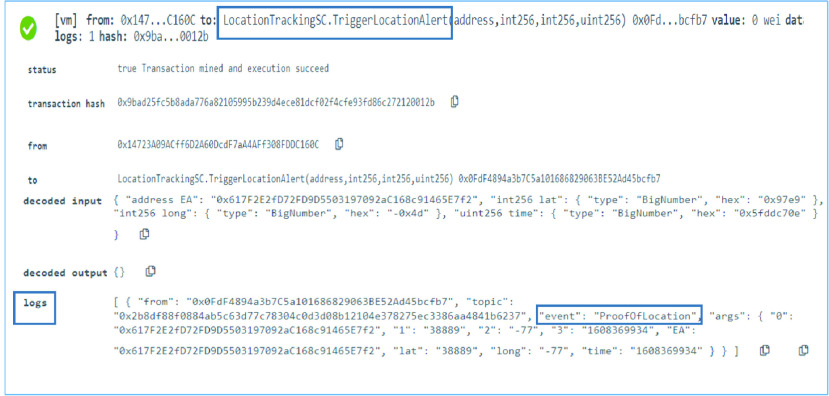
Logs showing a successful proof of location event.

A function called $TriggerLocationAlert$ notifies by triggering an event every time when the proof of location is sent to the blockchain. Figure 4 shows the latitude, longitude, user EA as well as the time successfully emitted as an event to all the participating entities as part of the immutable logs.

### B. Oracle SC: Oracle Registration

**FIGURE 5.**
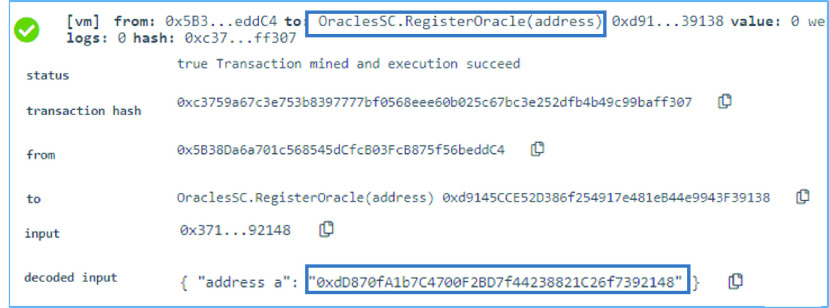
Logs of an oracle successfully registered in the Oracle SC.

Oracles need to be registered to execute functions call in the smart contract. Hence, the $RegisterOracle$ function is used to register the oracles as can be seen in figure 5. The registration is performed using the oracle’s EA. The EA is successfully added to the list of registered oracles mapping. Using this method, any oracle that tries to execute a function is first checked using an internal function in the Oracle SC to ensure that it is registered. The internal function returns a boolean which is the value mapped to the EA of the oracle at the time of the registration. ‘True’ is the boolean value mapped when registering the oracle. The registration can only be done by the owner of the Oracle SC. While testing, the Oracle SC owner holds the EA $0x5B38Da6a701c568545dCfcB03FcB875f56beddC4$ and the EA of the successfully registered oracle is $0xdD870fA1b7C4700F2BD7f44238821C26f7392148$ as can be seen in figure 5.

### C. Oracle SC: Oracle Revoking

**FIGURE 6.**
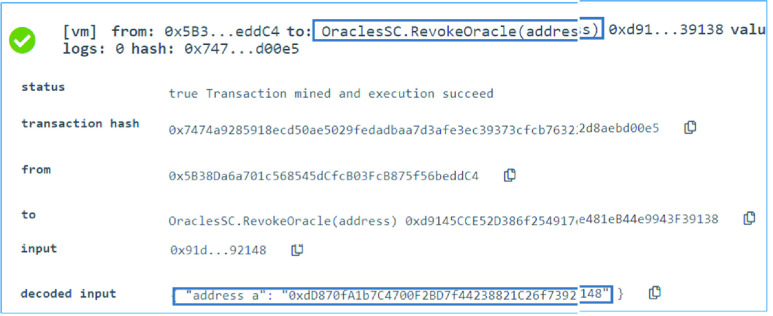
Logs of successfully revoking an oracle.

An oracle may no longer be trusted. Hence, a previously registered oracle with a boolean mapping of $true$ is revoked by changing its mapped value to $false$. Function $RevokeOracle$ in the $OracleSC$ is used to do so. The function once executed successfully changes the mapping and revokes an oracle. Figure 6 shows a successful execution of the function where the SC owner revoked the oracle with the EA $0xdD870fA1b7C4700F2BD7f44238821C26f7392148$ from its authorities.

### D. COVID-19 Notification SC: Broadcast COVID-19 Results

**FIGURE 7.**
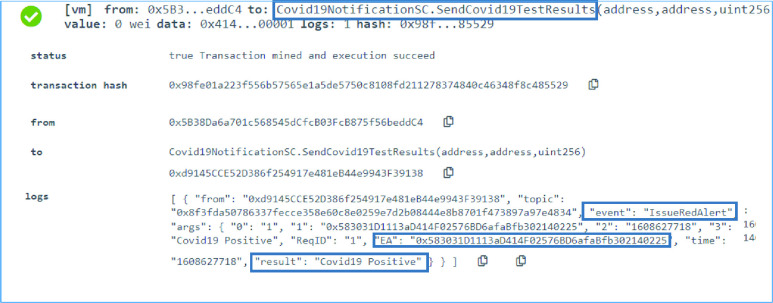
Logs of successfully emitting a red COVID-19 positive alert.

The testing center communicates through the COVID-19 Notification smart contract all the COVID-19 test results. The alerts are then emitted by the smart contract based on the results as discussed earlier in the previous sections. Hence, the testing was successfully done as seen in figure 7. A successful red, yellow and green alert is presented based on the passed result in the input. The EA of the test taker is also part of the emitted event. Moreover, if a yellow alert is emitted, the EA of the test taker as well as the direct contact the disease was passed from are both parts of the emitted event. The event also documents the time and the type of alert. The result is also part of the alert. Therefore, in figure 7 the result is $COVID-19\,\,Positive$ since the alert emitted is a red alert. The results $Contact$ or $Clear$ are for a yellow and green alert respectively. Furthermore, a unique request ID is also emitted with every event as shown in figure 7 where the request ID for this event is 1.

### E. COVID-19 Notification SC: Return Contact Tracing List Hash

**FIGURE 8.**
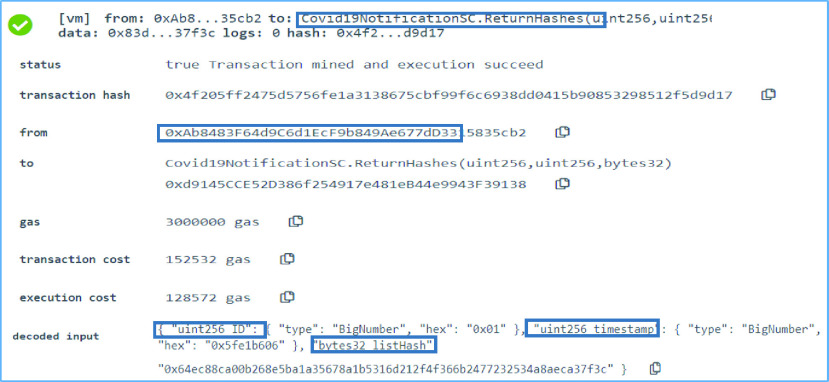
Logs showing a list hash successfully returned by a registered oracle.

The function $ReturnHashes$ in the COVID-19 Notification SC is used by the registered oracles to return the contact list hash. Upon being alerted with a yellow or red alert, the registered oracles execute the contact tracing algorithm and return the list hash to the smart contract. The registered oracle uses the request ID, request timestamp and the list hash when executing the function. In figure 8, the registered oracle 0xAb8483F64d9C6d1EcF9b849Ae677dD3315835cb2 entered the request ID which is 1 and the request timestamp 1608627718 as well as the list hash which is in bytes32 format as seen here, [“$0\times 64$”, “$0\times $EC”, “$0\times 88$”, “$0\times $CA”, “$0\times 00$”, “$0\times $B2”, “$0\times 68$”, “$0\times $E5”, “$0\times $BA”, “$0\times 1\text{A}$”, “$0\times 35$”, “$0\times 67$”, “$0\times 8\text{A}$”, “$0\times 1\text{B}$”, “$0\times 53$”, “$0\times 16$”, “$0\times $D2”, “$0\times 12$”, “$0\times $F4”, “$0\times $F3”, “$0\times 66$”, “$0\times $B2”, “$0\times 47$”, “$0\times 72$”, “$0\times 32$”, “$0\times 53$”, “$0\times 4\text{A}$”, “$0\times 8\text{A}$”, “$0\times $EC”, “$0\times $A3”, “$0\times 7\text{F}$”, “$0\times 3\text{C}$”]. The function is executed successfully and the hash is stored in the smart contract.

### F. COVID-19 Notification SC: Choosing the Winning Oracle

**FIGURE 9.**
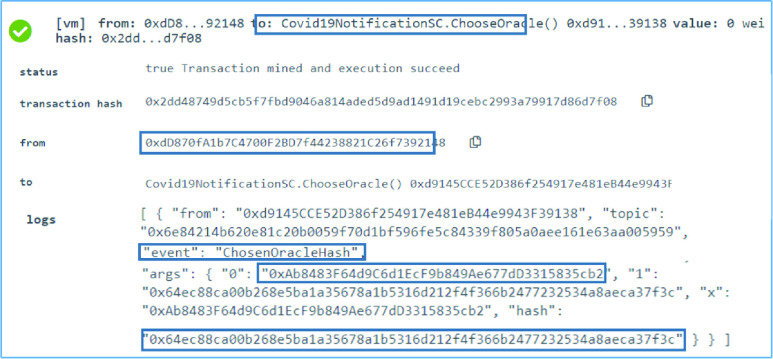
Logs showing a successful announcement of the chosen list hash and the winning registered oracle.

All registered oracles try to submit on time the hash list after executing the contact tracing algorithm. The timer oracle, holder of the EA 0xdD870fA1b7C4700F2BD7f44238821C 26f7392148 executes the $ChooseOracle$ function in the COVID-19 Notification SC when the time for the oracles to return the hashes is finished. The function checks the majority hash which is the most repeated hash and then returns the first registered oracle that has returned that hash. The function is executed successfully by the timer oracle. The chosen list hash and the oracle that submitted the hash are announced in an event for all the participating entities as can be seen in figure 9. The successful execution and testing of the $ChooseOracle$ function also indicate the successful execution of the internal function $findMajority$ function which is needed to find the majority hash that matches at least 50% of the submitted hashes in the list.

### G. COVID-19 Notification SC: Submit Contact Tracing List EAs

**FIGURE 10.**
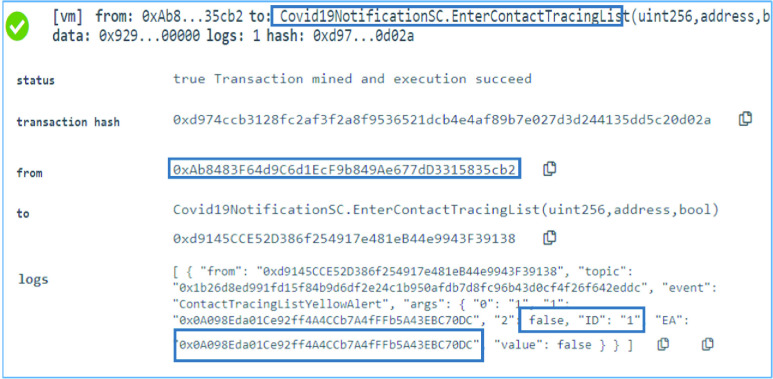
Logs indicating a successful entry of an EA in the contact tracing list.

The winning oracle then needs to submit the EAs of the list to the smart contract. This is done through the $EnterContactTracingList$ function. The function can only be executed by the winning oracle, otherwise, it would show an error and revert the contract state. The function takes the request ID, an EA, and a boolean which is only set to false to indicate the last item in the list. The EA submitted by the winning oracle is $0\times 0$A098Eda01Ce92ff4A4CCb7A4fFFb5A43 EBC70DC as can be seen in figure 10. This function emits an event that holds the details of the EA as well a contact tracing list yellow alert which can be used by the testing center for further action.

## V. Discussion

In this section, we evaluate our solution using four parameters; namely, security, cost, privacy, and generalization aspects to measure its security and privacy strengths and verify its affordability, feasibility, scope, and practicality.

### A. Security Analysis

Blockchain provides several intrinsic security features that are leveraged in our solution. Trusted and secure solutions can be built using its immutable and tamper-proof ledger. It eliminates exploits and vulnerabilities by incorporating authorization, availability, non-repudiation, accountability, integrity, and transparency. Each feature aforementioned is described below in detail with respect to our implemented solution.

Authorization is important to only allow designated entities to execute certain functions in the implementation. In our solution, every function in the smart contract can only be executed by a certain authorized entity. This is achieved through the usage of modifiers which help in checking the EA of the entity trying to execute the call and comparing it with the EA of the desired authorized entity. If the EA is not matching, then an error is shown and the smart contract state is revoked to the previous state.

Availability ensures a solution is robust, reliable, and trusted. A blockchain network is always available whenever needed. Any transaction can be executed at any time securely through the function calls in the smart contracts. Blockchain is also a decentralized and distributed ledger where each node has a local copy of all the transactions. Hence, the network is not prone to being a single point of failure or hacking, unlike centralized systems.

Moreover, Non-repudiation is an important and desirable feature that ensures no entity can deny its actions. Every transaction that is executed is part of the immutable logs and is digitally signed using the private key of the executor. Hence, no transaction on the chain is stored without details of the caller such as the Etherum address and the smart contract address. Furthermore, the digital signature clears any doubts related to the EA that executed the call as it implements accountability.

Furthermore, integrity is maintained through the tamper-proof logs and immutable information available on-chain. Any data stored on-chain cannot be altered, added to, or deleted. All the transactions that are created are stored and preserved. They can be used for history tracking and tracing as the logs are well-maintained and resilient.

On the other hand, another desirable feature of contact tracing solutions is privacy. Although Ethereum is a public blockchain network, several other permissioned blockchain networks that depend on channels, groups, and Membership Service Providers (MSP) can enable private communication between groups and entities. In the context of contact tracing, it is important to ensure that the privacy of the users using the contact tracing application is not imperiled. Consequently, our design associates the EAs of the users on-chain to their biometric data when registering at testing centers [22]. Furthermore, our solution uses digital medical passports [22] and gives the freedom to the user to allow access to their data and information. Without authorization from the data owner, the information cannot be accessed or used [22]. Hence, the data stored from the contact tracing cannot be used for purposes that are not transparent to the users. Transparency is easily achieved by using the decentralized blockchain ledger. All the transactions on-chain are accessible to all participants. The information is not used for purposes that are not known to the users beforehand.

### B. Cost Analysis

Any transaction executed on the ledger costs a certain fee. This fee is determined based on the current gas price (Gwei). Gwei is the price per unit of gas. Gas is used to determine how much is a transaction cost. As part of the logs, every executed transaction shows the transaction cost as well as the execution cost. The execution cost is a part of the transaction cost. In order to ensure that the solution is cost-efficient, an on-chain storage option is used to log transactions and alerts.

Gas prices keep fluctuating based on network congestion. Miners give high priority to transactions with higher gas prices. The more gas paid per transaction, the faster the miners would want to process it. Miners can decline a transaction if the gas price for it is too low and does not meet their set minimum threshold. In our cost analysis, we are using the gas prices found on the ETH Gas Station [31] on December 25, 2020. The prices for the fastest, fast, average, and cheap are 90, 84, 67, and 50 Gwei, respectively. In Table 1, we have used the cheap gas price of 50 Gwei in order to calculate the cost. Moreover, we have used a price of 300 USD for each Ether.

**TABLE 1:** Gas Cost in USD of Algorithm 7 for Multiple Array Elements

Number of Array Elements	Transaction Gas	Execution Gas	Cost USD
1	58508	39890	0.59835
2	50991	36132	0.764865
3	63072	42172	0.94608
4	79577	50425	1.193655
5	92628	56950	1.38942
6	101703	61488	1.525545
7	114806	68039	1.72209
8	129447	75360	1.941705
9	143916	82594	2.15874
10	158783	90028	2.381745

Table 1 shows the transaction cost as well as the execution cost in Gwei for algorithm 7 in the Notification SC. The table shows how as the number of elements increase in the array, the cost increases. Here, the number of elements represent the number of registered oracles that have responded before the deadline of a request. The cost is not negligible as can be seen from the table. However, the cost varies depending on the order of the array elements as well as their values. For instance, if 50% of the oracles responded with the same hash, the cost would be less compared to other scenarios where the oracles responding with different hashes are the first elements in the array. The cost in the table is expected since this algorithm has loops to choose the majority hash and element. Furthermore, the arrays are reset after every request. Once, the winning oracle is chosen the arrays are reset to use the same arrays for the next request ID.

A way of reducing the cost is possible by ensuring the registered oracles are honest and reliable. For instance, they need to be able to track that the current request ID the smart contract is taking hashes for is ID 1 for example. Therefore, they should all only reply back with the list hash for request ID 1 and should wait till the deadline is reached and the contract announces the new current ID is 2 for them to submit again. If this is ensured then there is no need to reset the array and the array can be looped from only the new elements added for ID 2 respectively. However, to ensure this is the case, the reputation of the oracles should be high and they should be trustworthy. Furthermore, watchdogs can also be used by other trusted and reputable registered oracles to ensure that all registered oracles are executing their tasks efficiently and honestly. This could greatly reduce the cost to only $ \$ $0.8964 for 10 elements in contract to 2.3817 as suggested by the table.

On the other hand, the minimum transaction size is approximately estimated as 100 bytes based on the Ethereum transaction’s logical structure in the Ethereum yellow paper [32]. The size depends on the ‘input data’ field available in the logs. The higher the value, the higher is the payload size. The payload data is formed by first calculating the keccak-256 hash of the function signature. The first 4 bytes of the function signature are the function selector that help in identifying each function. Then each argument is converted to Hex and padded into 32 bytes. There is no maximum for the payload size but it also depends on the gas consumption [33]. On-chain the logged transactions include the proof of locations whenever the distance is less than 2 meters, the list of contacts formulated by the oracles when a red or yellow alert is received, and the COVID-19 test results logged by the testing centers. So, on-chain data is mainly logs of the transactions.

The data stored off-chain is mainly the geolocation of the users stored on their devices. Storing a geolocation requires two floats where each one is of 4 bytes. Hence, one geolocation requires 8 bytes. The amount of storage required on a user’s device depends on how much the user socially interacts with others in a fixed time frame and how distant apart is the user in social gatherings and interactions. Hence, the minimum number of bytes stored on the device from processing the contact tracing geolocations are 8 bytes. The maximum depends on the phone’s storage space. However, a crowded scenario wherein the user is standing among 10 other people in less than 2m distance can generate a minimum of 88 bytes of data on the phone.

### C. Comparison With the Existing COVID-19 Contact Tracing Solutions

Blockchain as a breakthrough technology has aided a lot in the research against the COVID-19 pandemic. Our proposed solution is different from centralized contact tracing systems as blockchain and smart contracts are its core components, unlike traditional systems that depend on centralized servers such as the architecture mentioned in figure 1. Furthermore, Table 2 shows a comparison between the various COVID-19 contact tracing solutions that are based on blockchain. As can be seen from the table, there are different approaches and different features used in each solution. Some solutions proposed in [15], [30] depend on a hybrid approach that also requires the use of servers or trusted third parties. Although a solution proposed in [18] does not require the use of servers, it depends on formulas and probabilities to estimate the risk of having the COVID-19 infection. Hence, it is a risk notification and contact sharing system. The solution proposed in [16] depends on an artificial allocation mechanism using Internet of Things (IoT) devices to act as provers and witnesses. They must be incentivized to act honestly and be trustworthy. On the other hand, our proposed solution implemented in this research is a fully decentralized solution that depends on trusted on-chain oracles to execute the contact tracing algorithm based on the on-chain received COVID-19 results.

**TABLE 2:** Comparison With Other Covid-19 Contact Tracing Solutions

Solution	Communication	Features	Storage	Description	Blockchain Role	Smart Contracts	Blockchain Type
BeepTrace [16]	Bluetooth, GPS cellular, Wifi	Public key infrastructure	Uses third party servers for geomatching	Uses a certificate authority and a geo-solver. A hybrid system with servers and blockchains	Two blockchains: Tracing and notification	No	Permissioned
Covid-19 Risk Framework [19]	Bluetooth, GPS	location-based using GPS	Distributed blockchain database	Formulas are used to calculate the probability of infection ahead of time	Trace and notification	Yes	Permissionless
ByChain [17]	Bluetooth, GPS, LTE, Wifi	Zero-knowledge	Distributed blockchain	Bychain proposes a new location consensus based on the virtual electric field. They also use provers and witnesses.	Distributed database and a location-based consensus mechanisma	Yes	Permissionless
DIMY [31]	Bluetooth	Deffie-Hellman key generation and Bloom Filters	On user devices, on a server and blockchain	A hybrid system that uses a server along with the blockchain to preserve privacy using multiple methods	Risk analysis and notification	Chain codes	Permissioned
Our Proposed Solution	Bluetooth, GPS	Proof of locations	On user devices, blockchain, decentralized storage	A fully decentralized system that depends on on-chain oracles to execute contact tracing algorithms	Tracing, alerting notifications and an immutable database	Yes, made publicly available	Permissionless

### D. Generalization

Our solution is used to tackle an important and current problem which the world is facing due to the COVID-19 pandemic. However, although contact tracing is highly useful for COVID-19 to mitigate its aftermath effects, contact tracing can be used for any other contagious disease.

The presented blockchain-based solution can easily adapt to any other requirements of any contagious disease or application that requires contact tracing. Hence, this work is presented to help eradicate issues related to trust in contact tracing. Using blockchain and its intrinsic features as well as security characteristics make it ideal to establish trust, reliability, and a feasible solution. With the world now trying to lead a normal life in 2021, opening immigration as well as easing travel restrictions, contact tracing can greatly benefit to swiftly act if any case is determined as positive.

### E. Privacy Analysis

Contact tracing applications involve several parties and require the cooperation of many entities to be successful. It has shown promising results to prevent the spreading of the infectious COVID-19 [2]. For the users to consider using contact tracing applications willingly, they need to be offered transparency, privacy, and accountability. It is the right of users to be offered privacy for their shared data and information while using the contact tracing application. Also, they have the right to choose and decide how much their data can be disclosed. This is implemented to ensure that the information of users is not prone to hacking or abuse.

Our solution uses Self-Sovereign Identity (SSI) to ensure that each user owns their identity data and they do not rely on a management system. Blockchain-based identity systems manifest on digital identities to eradicate relying on centralized servers and third parties. It is a user-controlled data management system [34]. On the other hand, Christopher Allen identifies SSI as a way that makes users the administrator of their identities that may be stored across several locations upon their consent. He also adds that the identity information of a user can be asserted or certified by other groups as well [35].

Furthermore, in our design, an EA is not associated on-chain with any information that will reveal the true identity of the person. The information stored on-chain after registration as part of the medical digital passports only includes hashes. Hence, the true identity of a person is not revealed online and all transactions are made through only an electronic digital address. This kind of anonymity is similar to the anonymity used in Bitcoin where users are only known through electronic addresses [36]. Moreover, the proof of locations sent by the users on-chain are sent with a delay of 20 minutes to ensure the true location of a user is not known. This will preserve the privacy of users by maintaining their current location as confidential information.

## VI. Conclusion

In this paper, we have proposed a decentralized blockchain-based contact tracing solution to mitigate the spread of COVID-19. We showcased how blockchain-based immutable logs can add trust, transparency, and accountability features into COVID-19 contact tracing applications. In our approach, we leveraged blockchain’s built-in features to safeguard users’ information when using contact tracing applications. Our solution preserves users’ privacy by allowing them to choose when and to whom to share their information. We integrated the Ethereum blockchain with oracles to bridge the gap between the on-chain and off-chain data. We developed smart contracts, proposed eight algorithms, and discussed their full implementation and testing details. We evaluated the proposed approach using cost and security parameters that show it is affordable, practical, and secure enough against well-known attacks. The proposed solution also ensures privacy and can be easily adapted into different types of contact tracing applications as per their needs and requirements with minimal modifications. Hence, leveraging our solution for contact tracing applications can assist in curbing the spread of COVID-19.
